# Without getting under your skin: non-invasive stimulation activates the vagus nerve

**DOI:** 10.3389/fnins.2026.1829474

**Published:** 2026-06-10

**Authors:** Norianne T. Ingram, Colin Daniels, Nina Riggins, Jennifer R. Stevane, Peter S. Staats

**Affiliations:** 1 Vagus Nerve Society, Atlantic Beach, FL, United States; 2 Department of Mental Health, VA Puget Sound Healthcare System, Tacoma, WA, United States; 3 Headache Center of Excellence, Polytrauma, VA Palo Alto Healthcare System, Palo Alto, CA, United States; 4 Center for Holistic Surgery, Bozeman, MT, United States

**Keywords:** cervical nVNS, neuromodulation, neurophysiology, non-invasive, non-invasive vagus nerve stimulation, nVNS

## Abstract

Cervical non-invasive vagus nerve stimulation (nVNS) has emerged as a practical neuromodulation approach with FDA-cleared indications in primary headache disorders, yet skepticism persists over whether transcutaneous stimulation can reliably engage vagal fibers or whether observed benefits reflect nonspecific cervical activation. Here, we synthesize converging anatomical, biophysical, physiological, and clinical evidence demonstrating that nVNS does, in fact, activate vagal pathways without surgical implantation. We first review cervical vagus anatomy and the biophysical basis for target engagement, including ultrasound-measured nerve depth and multi-scale computational models showing that clinically relevant stimulation can recruit predominantly large myelinated vagal fibers. We then integrate mechanistic evidence across complementary modalities: functional imaging consistently modulates canonical vagal projection sites (including brainstem nuclei), electrophysiology demonstrates peripheral vagal recruitment and centrally transmitted evoked responses, immune studies reveal reproducible suppression of pro-inflammatory cytokines consistent with cholinergic anti-inflammatory reflex engagement, and autonomic biomarkers show shifts toward increased parasympathetic tone. Finally, we contextualize these mechanistic findings with sham-controlled randomized trials in cluster headache and migraine, where nVNS repeatedly outperforms sham for acute and preventive outcomes with a favorable safety profile. Together, these independent lines of evidence form a coherent mechanistic fingerprint that is difficult to reconcile with placebo or superficial muscle stimulation accounts. We conclude that nVNS provides a credible, scalable means of accessing vagal neurophysiology and represents a clinically validated, paradigm-shifting advance in bioelectronic medicine.

## 1 Introduction

The vagus nerve occupies a central position in human physiology. As the tenth cranial nerve, it serves as the principal parasympathetic conduit between brain and body, influencing autonomic tone, gastrointestinal function, cardiovascular regulation, pain perception, immune balance, and even emotional regulation through its projections into limbic structures (Staats et al., 2024).

Implantable vagus nerve stimulation (iVNS) has, for more than three decades, provided definitive clinical evidence that the vagus nerve can be therapeutically modulated. FDA approvals for epilepsy (1997), treatment-resistant depression (2005), and most recently rheumatoid arthritis (2025) established vagal stimulation as a validated therapeutic target. Yet, the surgical nature of implantation carries inherent limitations, including cost, risk of infection, and hardware complications (Spuck et al., 2010).

The advent of cervical non-invasive vagus nerve stimulation (nVNS) offers a safe and accessible alternative by delivering controlled electrical impulses transcutaneously at the neck (Gaul et al., 2016). Despite mounting evidence, some voices in the field have questioned whether non-invasive methods penetrate deeply enough to recruit vagal fibers, suggesting instead that therapeutic effects might reflect nonspecific cervical activation or placebo responses.

This position is increasingly untenable. A convergence of evidence across multiple independent domains—randomized controlled trials, neuroimaging, electrophysiology, immunology, and autonomic biomarkers—demonstrates unequivocally that nVNS activates vagal pathways. This review synthesizes that evidence. We first examine the anatomic and biophysical basis that supports cervical access to vagal fibers, then summarize experimental and clinical data spanning imaging, electrophysiology, immunology, and autonomic physiology. Finally, we contextualize nVNS within the broader neuromodulation landscape, drawing analogies to peripheral nerve stimulation paradigms that recalibrate central circuits through distant afferent input. These data establish nVNS not as an alternative to implantation, but as a paradigm-shifting advance in bioelectronic medicine.

## 2 Methods

### 2.1 Literature identification

Literature was identified through targeted searches of PubMed and Google Scholar, supplemented by reference tracing of key articles and prior reviews. Priority was given to peer-reviewed studies providing mechanistic insight (e.g., imaging, electrophysiology, immunologic, and autonomic measures) as well as randomized controlled trials evaluating clinical efficacy. Studies of auricular VNS were excluded to maintain focus on cervical stimulation, given differences in anatomy and fiber composition. The boundary between mechanistic and clinical evidence was defined by whether outcomes assessed physiological markers of vagal engagement versus patient-centered endpoints.

Study populations and sample sizes vary across trials, and a number of early studies employed open-label designs, introducing potential bias. Even among randomized controlled trials, heterogeneity in patient populations, endpoints, and dosing protocols may contribute to variability in reported outcomes.

### 2.2 Stimulation parameters

Across included studies, stimulation parameters were largely consistent, as most investigations utilized the same handheld cervical nVNS platform (gammaCore, electroCore, NJ). The gammaCore device delivers a 2-min train of a low-voltage (up to 24 V/60 mA), high-frequency (∼25 Hz) pulsed waveform with user-adjustable intensity. Accordingly, variability across studies primarily reflects differences in dosing protocols (e.g., number and timing of stimulations) rather than waveform characteristics such as pulse width or frequency. Factors such as electrode placement, contact pressure, and individual anatomical variability (e.g., neck circumference and tissue composition) may influence current delivery and target engagement and likely contribute to inter-individual differences in response.

An additional challenge specific to transcutaneous neuromodulation is the design of appropriate sham controls. Because cervical nVNS can produce perceptible sensations, including local tingling or muscle contraction, complete participant blinding is difficult to achieve. Sham conditions in major trials have typically employed low-intensity or altered waveform stimulation designed to mimic the sensory experience without engaging the vagus nerve, though the degree to which these approaches fully control for expectancy effects remains an area of ongoing discussion.

### 2.3 Statistical reporting

Statistical values are reported as presented in the original studies to preserve fidelity to source reporting; accordingly, formats may vary (e.g., exact *p*-values vs. threshold reporting).

## 3 Anatomy and biophysics

**FIGURE 1:**
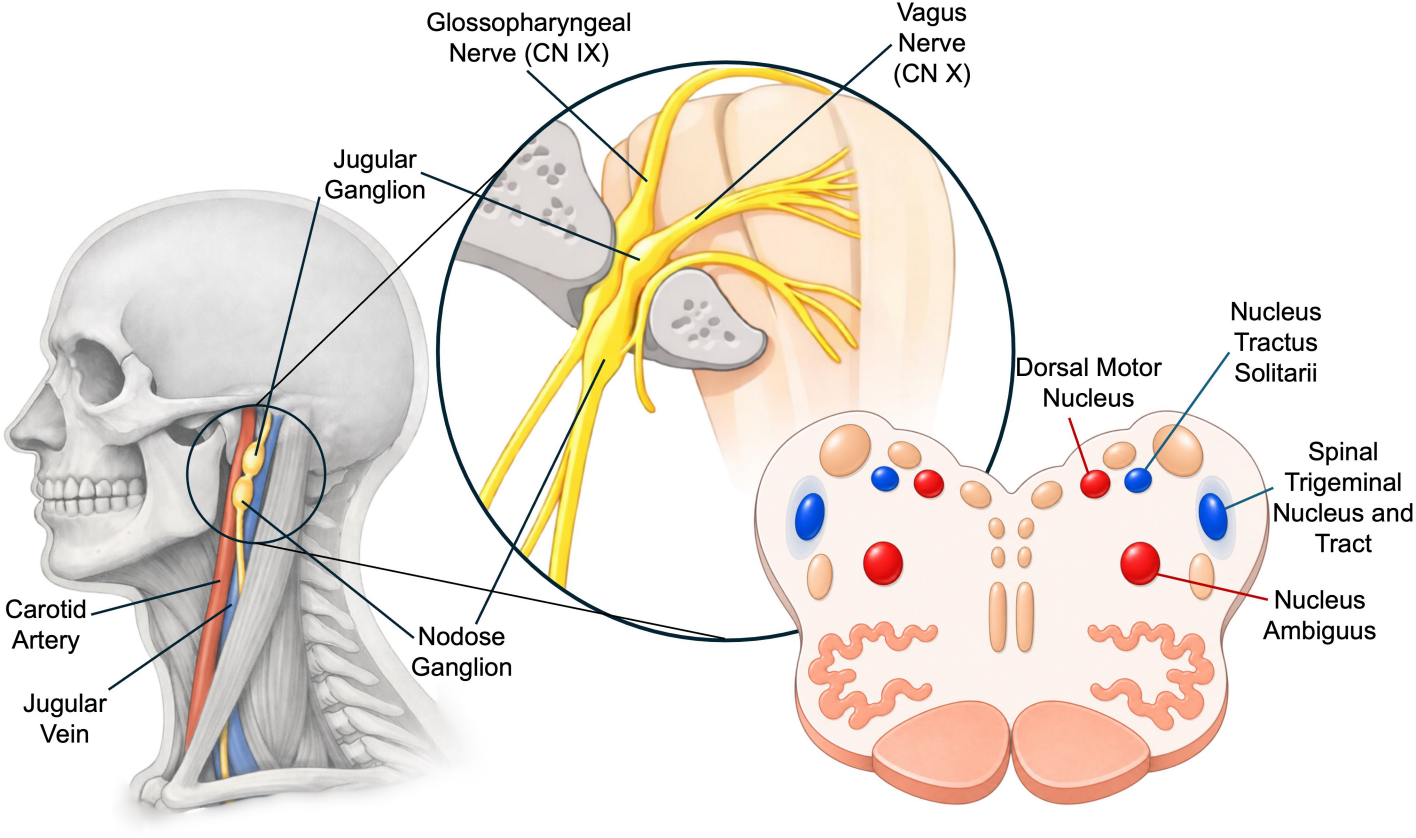
Cervical vagus nerve anatomy and afferent projections. The cell bodies of vagal afferent fibers reside in the jugular and nodose ganglia of the neck. Centrally, their axons project to the nucleus tractus solitarii (NTS) in the medulla. Peripherally, the cervical vagus nerve descends within the carotid sheath alongside the internal jugular vein and common carotid artery before branching toward visceral targets. Prior to extracervical branching, the right vagus contains up to ∼200,000 fibers and the left ∼100,000, of which approximately 80% are afferent, transmitting interoceptive sensory information from the viscera to the brainstem. Middle Panel image used under license from Kenhub GmbH. Illustrator: (Kim, 2026). Modified by N. Ingram.

The cell bodies of vagal afferent fibers are in the jugular and nodose ganglia of the neck (Figure 1). Their axons project centrally to the nucleus tractus solitarii (NTS) of the medulla, while peripherally they descend through the cervical region within the carotid sheath, alongside the internal jugular vein and common carotid artery (Berthoud and Neuhuber, 2000; Staats and Blake, 2024). Before branching toward their numerous visceral targets, the right vagus contains up to 200,000 fibers and the left approximately 100,000 (Havton et al., 2021). These fibers include both afferent and efferent pathways, with roughly 80% being afferent and responsible for transmitting interoceptive information from the viscera to the brain (Berthoud and Neuhuber, 2000; Groves and Brown, 2005). Accordingly, the present review focuses specifically on cervical nVNS; studies of auricular VNS, which targets a predominantly sensory cutaneous branch of the vagus, are not considered here due to fundamental differences in anatomy, fiber composition, and functional implications.

The cervical vagus nerve is an attractive neuromodulation target. At the level of the neck, it lies relatively close to the skin surface and has not yet branched extensively to its distal targets. Ultrasound measurements in healthy adults have shown the vagus nerve to be ∼1.3 cm (∼1/2 inch) beneath the skin (Lerman et al., 2016). Questions regarding whether nVNS can effectively reach this depth are addressed by analogy to other non-invasive stimulation modalities. For example, nerve conduction studies routinely stimulate tibial and peroneal nerves located up to ∼1 cm beneath the skin, and non-invasive peripheral nerve stimulation is widely used for motor rehabilitation and pain modulation. In the central nervous system, transcranial magnetic stimulation similarly demonstrates that non-invasive approaches can modulate both cortical and subcortical structures.

**FIGURE 2:**
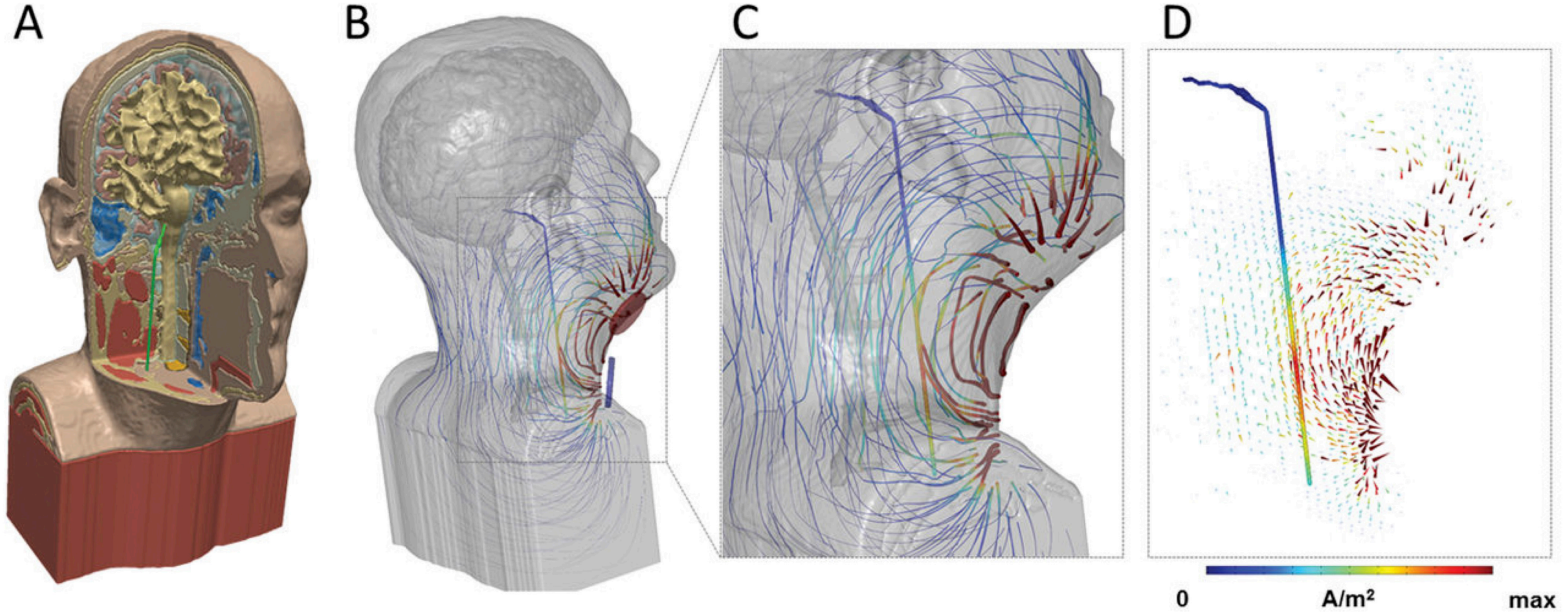
Computational modeling of cervical nVNS current spread predicts activation of vagal fibers. Multi-scale finite element modeling coupled with biophysical axon models demonstrates that clinically relevant cervical nVNS amplitudes preferentially activate large, myelinated A-fibers and larger B-fibers, while smaller B-fibers and unmyelinated C-fibers require substantially higher stimulation currents. Tissue inhomogeneity along the nerve alters local activating function profiles and shifts activation thresholds, but fiber diameter remains the primary determinant of excitability. These findings support selective engagement of fast-conducting vagal afferents during non-invasive stimulation. **(A)** MRI-derived anatomical model (bone, brain, muscle, and soft tissues) with the vagus nerve highlighted in green. **(B)** stimulation montage and resulting current-flow patterns, with a false-color map of local current density (>10 A/m^2^). **(C)** Zoomed inset of current flow around the vagus nerve (max ∼1.44 A/m^2^) for the same montage. **(D)** Arrow plots of gross and nerve-specific current densities; activation is predicted to depend on fiber type, under a quasi-static assumption with the anode in red and the cathode in blue to indicate instantaneous direction. Reproduced with permission from Mourdoukoutas et al. (2018).

Computational modeling studies further support the feasibility of cervical nVNS. Using anatomically realistic tissue parameters and a depth of ∼1.3 cm, these models demonstrate that transcutaneous stimulation can reach vagal fibers, particularly when waveform parameters and electrode placement are optimized (Mourdoukoutas et al., 2018; Figure 2). These findings establish the biophysical plausibility of target engagement under realistic anatomical conditions. While computational models do not constitute direct empirical evidence of nerve activation, this framework is supported by subsequent electrophysiological findings (e.g., eCAPs, vSEPs) demonstrating functional vagal activation.

Mechanistically, the effects of nVNS are thought to be primarily mediated by afferent fibers projecting to the brainstem. Stimulation of these fibers activates the NTS and downstream nuclei, including the locus coeruleus (LC), a principal source of norepinephrine in the brain. LC activation is implicated in regulating arousal, attention, and consciousness (Khroud et al., 2002).

Although afferents predominate, efferent pathways also traverse the cervical vagus and warrant consideration. These fibers originate in the dorsal motor nucleus of the vagus (DMV) and nucleus ambiguus (AMB) and include both preganglionic parasympathetic projections and branchial motor efferents (Groves and Brown, 2005). Notably, motor fibers contributing to the recurrent laryngeal nerve innervate laryngeal musculature, providing a plausible substrate for stimulation-related side effects such as voice alteration or hoarseness, consistent with recruitment of large myelinated Aα fibers (Berthoud and Neuhuber, 2000).

Importantly, the electrophysiological properties of vagal efferents may vary across species. While many DMV-derived fibers are unmyelinated in small animal models, studies in large mammals demonstrate conduction velocities consistent with myelinated fibers, suggesting the presence of B- or Aδ-type efferents that may be more readily recruited by electrical stimulation (Booth et al., 2021). While efferent activation may occur at clinically relevant intensities, the predominance and lower activation thresholds of large-diameter afferents remain the most parsimonious explanation for nVNS effects.

Taken together, the superficial anatomical position of the cervical vagus nerve, its high fiber density, and the central physiological role of its afferent projections provide a strong biophysical rationale for non-invasive cervical stimulation as a reliable means of engaging vagal pathways.

## 4 Physiological evidence of non-invasive activation

### 4.1 Functional imaging

Functional magnetic resonance imaging (fMRI) measures changes in blood flow and oxygen consumption in the brain and is correlated with neuronal activity. fMRI studies have demonstrated that nVNS modulates brain regions associated with canonical vagal projections, including the NTS, LC, dorsal raphe nucleus, insular cortex, and cingulate cortex (Frangos and Komisaruk, 2017). These findings mirror those reported with implanted VNS, suggesting that non-invasive stimulation engages the same central autonomic circuits (Henry et al., 1998).

Positron emission tomography (PET) is used to measure metabolic activity at a cellular level and provides complementary evidence. PET studies demonstrate that nVNS modulates limbic–cingulate–prefrontal responses: in trauma-exposed adults without post-traumatic stress disorder (PTSD), nVNS reduced activation in medial/orbitofrontal, temporal, insular, and anterior cingulate regions compared to sham stimulation; in adults with PTSD, nVNS increased anterior cingulate and hippocampal activation (Wittbrodt et al., 2020). These PET findings align with central autonomic network engagement by nVNS.

Together, fMRI and PET imaging confirm that nVNS does not act locally at the skin or muscle level, but instead produces reproducible modulation of central vagal pathways deep in the brain.

### 4.2 Electrophysiology

Electrophysiological measures prove that nVNS directly engages vagal fibers. At the peripheral level, evoked compound action potentials (eCAPs) have been recorded from the cervical vagus nerve during nVNS (30 V). The conduction velocities were consistent with Aδ fibers (∼5 m/s), B fibers (∼3.2 m/s), and at higher stimulation intensities (75 V), triphasic action potentials characteristic of C fibers (0.88–1.17 m/s) (Bu et al., 2021).

While Bu et al. (2021) show that nVNS can induce vagal fibers to generate action potentials, it could still be argued that those signals do not reach central structures in the brain. However, Nonis et al. (2017) measured vagal somatosensory evoked potentials (vSEPs) in 80% of healthy subjects during cervical nVNS at 15 V. vSEPs are electrical brain impulses recorded through the scalp and are generated from the stimulation of the vagus nerve; they are observed during iVNS. When Nonis et al. (2017) applied the same stimulation to the sternocleidomastoid muscle (SCM), vSEPs were not observed demonstrating that their findings are specific to nVNS activation of the vagus nerve. Together, these recordings confirm that nVNS directly recruits vagal fibers and generates signals that are transmitted to the central structures that are involved in vagal processing.

**FIGURE 3:**
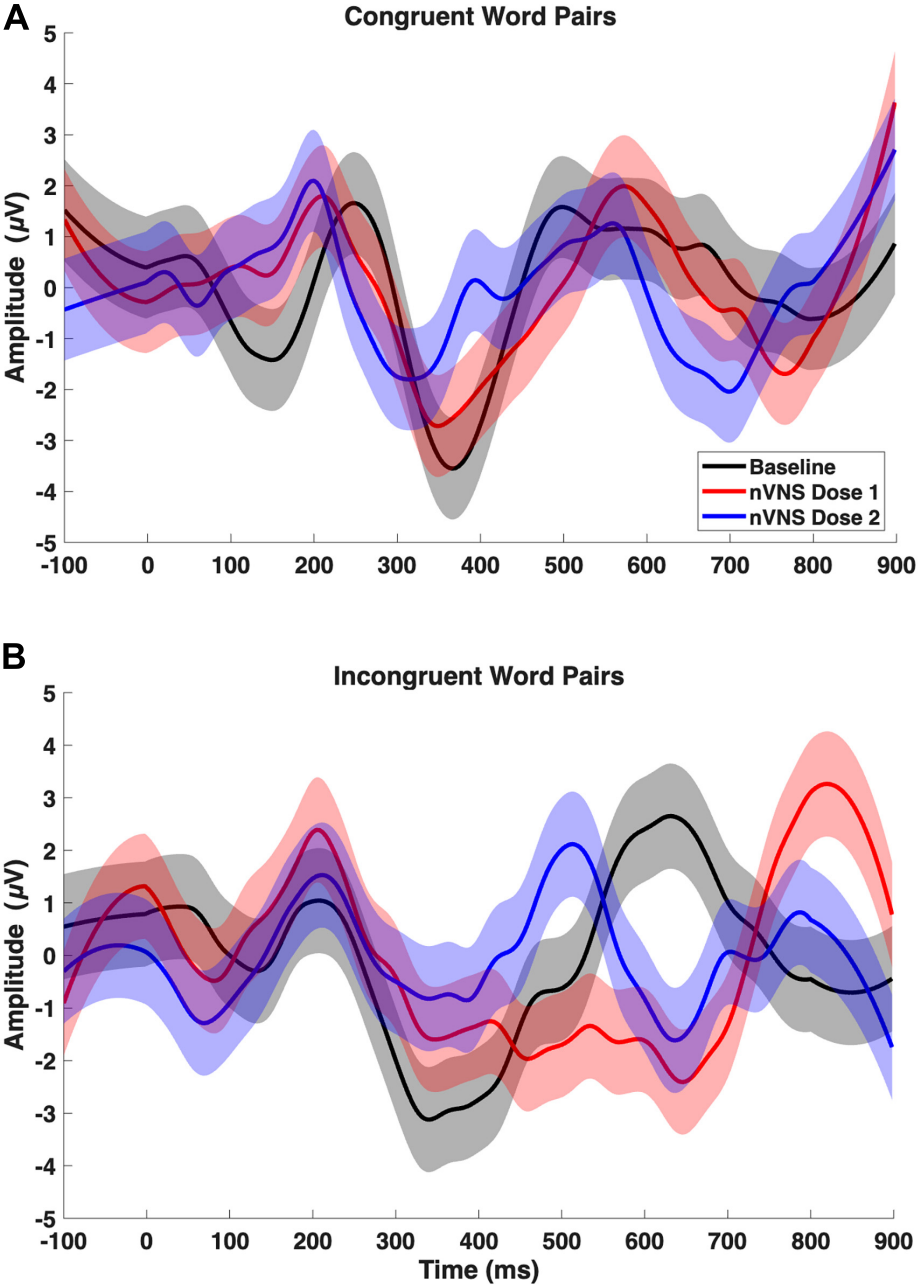
nVNS alters cortical processing in auditory task ERPs. EEGs were collected with NeuroCatch Cognitive Evaluation Platform (NeuroCatch, Inc., British Columbia). This is a 3-electrode system that records auditory processing while a subject identifies congruent (36/session) and incongruent (36/session) word pairs. In these example traces baseline (black) was recorded prior to nVNS exposure for the day. Six minutes of nVNS was delivered to the subject prior to repeating word pair tasks (red). The subject was then exposed to eight more minutes of nVNS (blue). Data are presented as mean ± SD. For congruent word pairs **(A)**, the amplitude and latency of the N400 decreased after nVNS suggesting faster processing and decreased cognitive load. The effects of nVNS on identifying incongruent word pairs are more involved **(B)**. N400 decreases with each dose of nVNS. Later components related to cortical processes show greater changes from baseline. Data provided by Daniels (unpublished data). Representative data from a single subject.

Building to the cortical level, electroencephalography (EEG) and event-related potential (ERP) studies provide further evidence of vagal modulation of brain activity during nVNS. In a controlled trial, cervical nVNS reliably modulated both early and late ERP components (Lewine et al., 2019). Stimulation enhanced the N100, an index of early cortical sensory processing, by improving auditory sensory gating. It also increased the amplitude of the P300b by ∼40%. The P300b emerges around 300 ms and reflects higher-order attentional processing. The P300b effects persisted for at least two hours post-stimulation; sham stimulation produced no such changes. Work to understand nVNS effects on later ERP components is underway (Figure 3). Again, cortical activity after nVNS stimulation closely mirrors that reported with iVNS (Workewych et al., 2020).

The electrophysiological data reviewed here demonstrate that nVNS drives active vagal fiber signaling (eCAPs) and induces measurable effects in the higher-order cognitive functions of central nervous system structures (vSEP and ERPs) like those seen with iVNS. Sham stimulation does not induce the same effects which supports an argument for specific activation of the vagus nerve by nVNS.

### 4.3 Immunologic responses

The vagus nerve exerts potent control over inflammation via the cholinergic anti-inflammatory pathway (CAP). In this framework, vagal efferents suppress pro-inflammatory cytokine release, including tumor necrosis factor-α (TNF-α), interleukin-1β (IL-1β), and IL-6 through α7 nicotinic acetylcholine receptors on macrophages (Pavlov et al., 2003).

In addition to this efferent mechanism, growing evidence suggests the anti-inflammatory effects of VNS involve a more complex interaction between vagal and sympathetic systems. Stimulation of vagal afferents can engage central autonomic circuits, including the NTS, which in turn activate brainstem pre-sympathetic neurons projecting to thoracolumbar sympathetic outflow. Experimental evidence in rodents shows that these effects are abolished by sectioning the splanchnic sympathetic nerves, indicating that sympathetic pathways may serve as the final effector of this reflex (Komegae et al., 2018). Together, these findings support a model in which vagal afferent activation modulates immune responses indirectly via reflex recruitment of sympathetic anti-inflammatory pathways.

Regardless of whether these effects are mediated by efferent, afferent, or integrated vagal–sympathetic circuits, the observation that cervical nVNS reproducibly alters cytokine profiles in humans provides direct mechanistic evidence that nVNS penetrates sufficiently to recruit vagal fibers.

#### 4.3.1 Cytokine modulation in healthy humans

The first sham-controlled demonstration came from Lerman et al. (2016), who conducted a randomized, blinded trial in healthy adults. Subjects received three 2-min nVNS stimulations over a 24-h period. Twenty-four hours after the first stimulation, individuals who received nVNS exhibited significant reductions in pro-inflammatory cytokines (IL-1β, TNF-α, IL-8, monocyte chemoattractant protein-1, macrophage inflammatory protein-1α) and an increase in the anti-inflammatory cytokine IL-10 compared with sham (*p* < 0.05). These findings provided the first human evidence that nVNS activates the CAP with effects not attributable to sham stimulation (Lerman et al., 2016).

#### 4.3.2 Autoimmune and inflammatory diseases

Clinical extensions into autoimmune disease show consistent immune signatures. In human patients with psoriatic arthritis and ankylosing spondylitis, both conditions characterized by chronic inflammation, five days of nVNS (2-min stimulation, 3× per day) reduced disease activity and lowered C-reactive protein (CRP), interferon-γ (IFN-γ), and IL-8 (Brock et al., 2021).

Similarly, in hospitalized patients with acute COVID-19, the SAVIOR-I trial demonstrated that five days of nVNS (two consecutive 2-min stimulations, 3× per day) plus standard of care produced significantly greater reductions in CRP (*p* = 0.015) and procalcitonin (*p* = 0.015) compared with control (Tornero et al., 2022).

Finally, in a double-blind sham-controlled study of patients with Sjögren’s syndrome (an autoimmune disease which affects moisture-producing glands), Tarn et al. (2023) reported significant improvements in fatigue (*p* = 0.02) and cognition (*p* = 0.02) with nVNS administered twice daily for 54 days. EEG correlates suggested restoration of cholinergic signaling, linking symptomatic benefit to vagal CAP activation (Tarn et al., 2023).

These findings extend CAP activation to systemic inflammatory disorders and infectious disease.

#### 4.3.3 Neuroinflammation in migraine and TBI

Neuroinflammation contributes significantly to the development of neurological diseases. In clinical studies of migraine, IL-1β rose in sham-treated patients but remained stable in nVNS-treated patients after two months (*p* < 0.05) (Chaudhry et al., 2019).

In traumatic brain injury, preclinical evidence further supports this mechanism. A mouse study demonstrated that prophylactic nVNS suppressed IL-1β signaling (*p* < 0.01) and improved behavioral recovery (*p* < 0.05) (Morais et al., 2024).

Emerging human data suggest similar translational potential. In a pilot study of patients with acute stroke, individuals who received 14 doses of nVNS within 6 h of symptom onset showed reduced infarct growth compared to those receiving sham stimulation (63.3% vs. 184.2%; *p* = 0.109) (Arsava et al., 2022). The authors proposed that this effect may be mediated through the suppression of neuroinflammation.

In a larger human cohort, a retrospective observational study of > 100 patients with persistent post-concussion symptoms found that adjunctive nVNS was associated with improvement across cognitive, affective, vestibular, and somatic symptom domains, independent of time since injury (Ament et al., 2025).

Together, these findings support the hypothesis that nVNS may attenuate maladaptive neuroimmune and neurophysiologic cascades in neurological injury, while motivating prospective biomarker-informed trials.

#### 4.3.4 Stress-induced inflammation

In PTSD, stress-induced cytokine release provides a direct biomarker of vagal modulation. In a randomized, sham-controlled trial, patients with PTSD who were re-exposed to traumatic cues exhibited increases in proinflammatory cytokines IL-6 and IFN-γ under sham stimulation. These increases were attenuated in patients who received nVNS immediately after re-exposure (*p* < 0.05) (Bremner et al., 2020).

In the same cohort, patients receiving active nVNS also demonstrated significantly greater reductions in PTSD symptom severity (PCL-5 reduction, *p* = 0.013; HAM-A reduction, *p* < 0.05) (Bremner et al., 2021).

Because stress-induced cytokine release is vagally regulated, these findings strongly support direct vagal recruitment by nVNS in humans.

#### 4.3.5 Synthesis: cytokines as evidence of vagal engagement

Across independent studies in healthy adults, autoimmune disease, infection, migraine, and PTSD, nVNS consistently suppresses pro-inflammatory cytokines (IL-1β, IL-6, TNF-α, IFN-γ) and, in some cases, enhances anti-inflammatory cytokines. These shifts are unlikely to be explained by superficial muscle stimulation or placebo effects, but instead are consistent with activation of the cholinergic anti-inflammatory reflex. In this context, cytokine modulation provides functional evidence of non-invasive activation of vagal pathways, reinforcing convergent findings from anatomy, imaging, and electrophysiology.

### 4.4 Autonomic and cardiovascular evidence

Cardiovascular biomarkers provide a direct and measurable physiologic readout of sympathetic vs. parasympathetic balance, as the degree of vagal nerve activation sets vascular tone and other readily accessible cardiac parameters. Across studies in healthy individuals and in patients with PTSD, nVNS consistently increases parasympathetic tone, attenuates sympathetic arousal, and restores autonomic balance as measured by cardiovascular indices. Importantly, these parasympathetic effects cannot be explained by superficial cervical stimulation or placebo, providing strong evidence that nVNS shifts cardiovascular regulation toward true vagal dominance.

In a sham-controlled trial evaluating individuals with PTSD, patients who received active nVNS experienced decreased heart rates, increased peripheral vasodilation (photoplethysmogram amplitude), and improved vascular compliance (prolonged pulse arrival time) compared to those who received sham stimulation (Gurel et al., 2020). Gurel et al. (2020) also observed increases in long-term heart rate variability (HRV), which were not seen in the sham stimulation group. While HRV is widely used as a non-invasive correlate of cardiac vagal modulation (Shaffer and Ginsberg, 2017), it should be noted that commonly used HRV metrics—particularly respiratory-related HRV (respiratory sinus arrhythmia)—primarily reflect phasic modulation of cardiac autonomic activity rather than a direct measure of overall vagal tone, and are influenced by respiratory, mechanical, and sympathetic factors (Menuet et al., 2025).

Using advanced signal processing, Gazi et al. (2021) demonstrated that nVNS inhibited stress-induced increases in the reciprocal of pulse transit time (1/PTT), a surrogate marker of arterial stiffness and blood pressure load (Gazi et al., 2021). These effects are consistent with parasympathetic activation and a shift from a sympathetic-dominant cardiovascular profile; they cannot be explained by superficial stimulation.

Finally, similar increases in cardiac vagal tone were seen in healthy adults who received bilateral nVNS. Cardiac vagal tone is a real-time measurement of efferent vagal drive to the heart. This cohort also experienced a reduction in the inflammatory marker TNF-α (Brock et al., 2017).

Cardiovascular outcomes provide real-time, physiologic evidence that nVNS engages the vagus nerve. Increases in long term HRV, suppression of sympathetic arousal, and improved vascular compliance all represent canonical vagal outputs. These effects cannot be attributed to nonspecific neck muscle stimulation, but instead reflect direct vagal efferent activation, reinforcing the imaging, electrophysiologic, and functional immune evidence reviewed above.

### 4.5 Convergence of physiological evidence

Taken together, the physiological evidence across multiple domains demonstrates unequivocally that nVNS activates the vagus nerve. Functional imaging shows recruitment of canonical vagal projection sites in the brainstem and forebrain (Frangos and Komisaruk, 2017; Yakunina et al., 2017; Wittbrodt et al., 2020). Electrophysiological recordings reveal evoked compound action potentials in cervical vagal fibers and cortical event-related potentials consistent with successful vagal afferent signaling (Lewine et al., 2019; Bu et al., 2021). Immunologic studies document activation of the cholinergic anti-inflammatory reflex in both healthy volunteers and in patients with inflammatory conditions. They show reproducible suppression of pro-inflammatory cytokines and enhancement of anti-inflammatory signaling (Pavlov et al., 2003; Lerman et al., 2016; Chaudhry et al., 2019; Bremner et al., 2020; Brock et al., 2021; Tornero et al., 2022; Tarn et al., 2023; Morais et al., 2024). Autonomic and cardiovascular outcomes (increased HRV, suppression of sympathetic reactivity during stress, and improved vascular compliance) provide real-time physiological confirmation of vagal efferent recruitment (Bremner et al., 2020; Gurel et al., 2020; Brock et al., 2021; Gazi et al., 2021).

The reproducibility of these effects across independent methodologies—neuroimaging, electrophysiology, immune modulation, and autonomic physiology—makes alternative explanations such as superficial cervical activation or placebo responses untenable. Instead, the cumulative data converge on a single conclusion: non-invasive cervical stimulation reliably engages the vagus nerve and produces downstream physiological effects indistinguishable from those of implanted stimulation.

## 5 Clinical evidence from randomized controlled trials

### 5.1 Pathophysiology of primary headache disorders

A primary headache is defined as a headache that is not caused by another condition, disorder, or injury. Here we review the pathophysiology of the primary headache conditions: migraine and trigeminal autonomic cephalalgias (TACs) including cluster headache. While International Classification of Headache Disorders (3rd edition) classifies these as separate conditions, several shared underlying mechanisms make nVNS a strong candidate therapy for all.

(1) Autonomic symptoms are key elements in TACs and are well known to present throughout migraine phases suggesting that dysfunction of the autonomic nervous system is a shared fundamental feature in these conditions.

(2) The initiation of head pain in migraine and TACs is thought to be related to the constriction of the cerebrovasculature. Constriction activates the trigeminovascular reflex which causes the release of neuroactive, inflammatory peptides like substance *P* and calcitonin gene-related protein. These inflammatory peptides prime the nervous system to become more sensitive to pain stimuli (Edvinsson, 2001). nVNS suppresses the release of these peptides in preclinical models (Liu et al., 2022).

(3) Head and neck pain is signaled to the brain through the spinal trigeminal nucleus in the brainstem: fMRI recordings show that nVNS reduces activity in this nucleus (Frangos and Komisaruk, 2017).

### 5.2 Cluster headache and other TACs

Fairly recently, cervical nVNS became the first and only therapy to gain FDA clearances for both the acute (2017) and preventive (2018) treatment of cluster headache. Cluster headache, often termed the “*suicide headache*” due to its excruciating intensity, represents one of the most disabling primary headache disorders.

**FIGURE 4:**
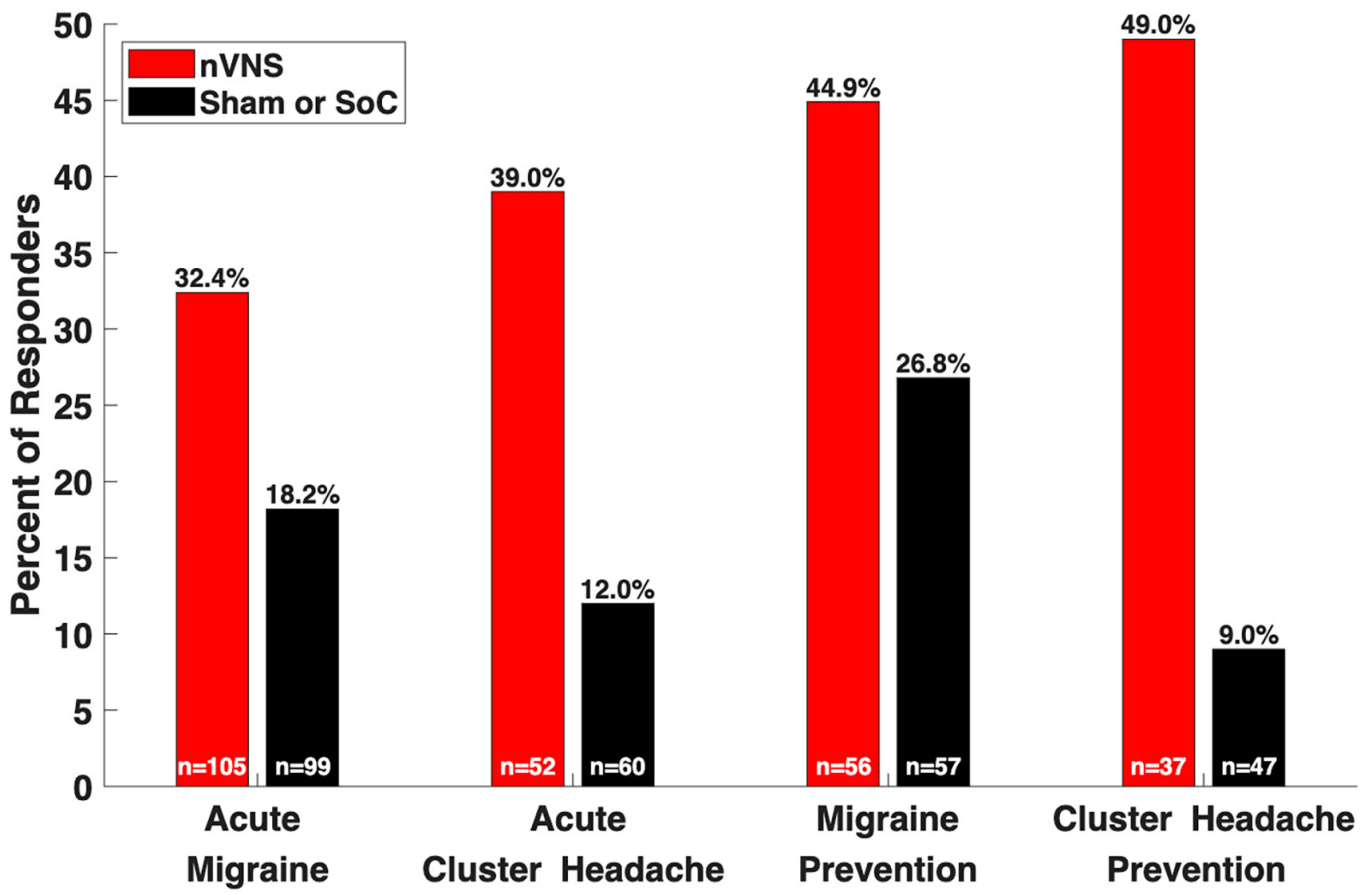
Acute and preventative nVNS responders with migraine or cluster headache. Percentage of nVNS responders by indication and definition: (1) Acute migraine: percentage of patients reaching pain free status at 120 min in 50% or more of migraine headaches treated (Tassorelli et al., 2018). (2) Acute cluster headache: percentage of patients reaching pain free status at 15 min in 50% or more of headaches treated (de Coo et al., 2019). (3) Migraine prevention: percentage of patients that reduced the monthly migraine frequency by 50% (Najib et al., 2022). (4) Cluster headache prevention: percentage of patients that reduced frequency of headaches by 50% or more from baseline (Gaul et al., 2016).

Randomized controlled trials have demonstrated that cervical nVNS significantly reduces both attack frequency and acute pain burden compared to sham stimulation. In the PREVA trial, patients receiving adjunctive nVNS experienced a 43% reduction in weekly attack frequency, compared to a 13% reduction in the sham group. The proportion of patients achieving ≥ 50% reduction in attack frequency was significantly higher in the nVNS arm (48.6% vs. 8.5%, *p* < 0.001) (Gaul et al., 2016). Parallel acute treatment trials (ACT1 and ACT2) further demonstrated that nVNS produced a higher proportion of pain-free responses within 15 min of nVNS compared to sham with the strongest effects observed in episodic cluster headache (Figure 4; pooled data for episodic cluster headache: 39% vs. 12%, *p* < 0.01) (Silberstein et al., 2016b; Goadsby et al., 2018; de Coo et al., 2019). Importantly, nVNS provided meaningful relief with an excellent safety and tolerability profile—no serious device-related adverse events were reported, and most patients described stimulation as mild and transient.

More recently, FDA clearances for hemicrania continua and paroxysmal hemicrania were added in 2020. While these TACs are rarer than cluster headache, a recent longitudinal case series reported by Fernandes et al. (2025) found that 65% of TAC patients found nVNS to be a meaningful tool in managing their condition, and these patients used nVNS for a median time of 47 months (interquartile range = 18–66 months). nVNS was noted to be especially effective as a preventative treatment, and the authors note that nVNS should be used as an earlier treatment option (Fernandes et al., 2025).

While clinical benefits in cluster headache were first appreciated for nVNS, new data is beginning to demonstrate similar efficacy for iVNS therapy in these patients (van Tilborg et al., 2025). iVNS findings strongly support shared therapeutic action through vagal activation.

Taken together, these findings firmly establish that non-invasive stimulation of the cervical vagus nerve provides robust therapeutic benefit in cluster headache, reinforcing the conclusion that nVNS engages vagal pathways comparably to implanted stimulation.

### 5.3 Migraine

nVNS has also been extensively studied in migraine, leading to FDA clearance for both acute (2018) and preventive (2020) indications in adults and adolescents 12 and older. Sham-controlled acute treatment trials, most notably the multicenter PRESTO study, demonstrated that nVNS significantly increased the proportion of patients achieving pain relief and pain freedom within two hours of stimulation compared with sham (Tassorelli et al., 2018). In PRESTO, three times as many patients who received nVNS were pain free by 30 min compared to the sham-controlled group (*p* < 0.012). Importantly, these effects were consistent across multiple migraine attacks, underscoring that benefit could not be explained by placebo or expectancy effects.

Preventive trials further support the efficacy of nVNS. In chronic migraine, the EVENT study demonstrated that prophylactic nVNS reduced headache days during open-label treatment, with excellent safety and tolerability (Silberstein et al., 2016a). In episodic migraine, the PREMIUM I trial showed that patients with good adherence (> 67% compliance) experienced a significant reduction in monthly migraine days compared with sham (2.27 vs. 1.53; *p* = 0.043) (Diener et al., 2019). The subsequent PREMIUM II trial again indicated preventive benefit: the ≥ 50% responder rate was higher with nVNS than sham (44.9% vs. 26.8%; *p* = 0.048), with the strongest effects observed in patients with migraine with aura (Najib et al., 2022). Taken together, these findings highlight the preventive potential of cervical nVNS, particularly when treatment adherence is maintained (Figure 4).

Additional studies have broadened the scope of evidence. In menstrual migraine, open-label trials showed that perimenstrual nVNS reduced attack frequency, analgesic use, and disability scores without safety concerns (Grazzi et al., 2016). In vestibular migraine, a retrospective study found that nVNS rapidly relieved vertigo and headache intensity all but 1 patient within 15 min of stimulation (Beh and Friedman, 2019). Pediatric data also support safety: in an adolescent pilot study, nearly half of treated attacks responded to nVNS with no device-related adverse events (Grazzi et al., 2017). Across all migraine populations, adverse events were consistently mild and transient—most often skin or neck sensations—while no serious device-related adverse events were reported.

### 5.4 Safety and tolerability in clinical practice

Across clinical trials, cervical nVNS demonstrates a favorable safety and tolerability profile. Reported adverse events are generally mild and transient, most commonly including local skin irritation, tingling at the stimulation site, and occasional neck muscle contraction. Transient voice alteration or hoarseness has been reported infrequently, consistent with potential recruitment of adjacent motor fibers (U.S. Food and Drug Administration, 2015). Rates of discontinuation due to adverse events are low across randomized controlled trials, and no serious device-related adverse events have been observed (Gaul et al., 2016; Silberstein et al., 2016a; Tornero et al., 2022).

This favorable safety profile extends to younger populations. Cervical nVNS is cleared for use in patients aged 12 years and older, and preliminary clinical data in adolescents aged 13–18 years demonstrate good tolerability with no device-related adverse events (Grazzi et al., 2017).

This safety profile represents a key advantage of non-invasive approaches compared to implanted VNS systems and supports the feasibility of repeated, at-home use. Importantly, the nature of reported effects is consistent with localized and controlled activation of cervical vagal pathways rather than nonspecific or widespread off-target stimulation.

### 5.5 Mechanistic implications of sham-controlled evidence

Across TACs and migraine, the consistent demonstration of superiority of cervical nVNS over sham stimulation provides critical mechanistic insight: the therapeutic effects cannot be explained by placebo, expectancy, or nonspecific cutaneous stimulation alone. Instead, the sham-controlled design strongly indicates that clinical benefit arises from effective engagement of the vagus nerve. The convergence of acute and preventive efficacy across multiple independent randomized trials and case series highlights that nVNS activates the vagal afferent pathways that modulate trigeminal-autonomic circuits and central pain networks. Collectively, these findings provide compelling evidence that nVNS is a true non-invasive means of accessing vagal mechanisms to produce clinically meaningful neurological effects.

## 6 Future directions

The vagus nerve provides extensive bidirectional communication between the brain and peripheral organs, spanning cortical and brainstem circuits as well as visceral systems. Although the clinical evidence base for cervical nVNS is already substantial, additional therapeutic applications are under active investigation, particularly in conditions characterized by dysregulation of autonomic, inflammatory, or central neural circuits. These emerging indications represent a natural extension of vagal neuromodulation. A critical next step in translating these applications will be the development of condition-specific dosing strategies, as optimal stimulation parameters—including timing, frequency, and cumulative exposure—are likely to vary across disease states. Below, we highlight representative areas of ongoing investigation and their mechanistic rationale.

Inflammatory and autoimmune disorders: Early clinical studies in patients with rheumatoid arthritis and primary Sjögren’s syndrome demonstrate that nVNS can suppress pro-inflammatory cytokine signaling while providing meaningful symptomatic relief (Drewes et al., 2021; Tarn et al., 2023). These findings provide a strong rationale for extending investigation to other conditions characterized by immune dysregulation, including systemic lupus erythematosus, psoriasis, asthma, and long COVID, in which dysregulated immune responses are central to disease pathology.

Neuropsychiatric and cognitive applications: Preclinical and early human data suggest that nVNS modulates limbic and prefrontal circuits involved in emotional regulation, raising the possibility of therapeutic benefit in depression, PTSD, and anxiety (Bremner et al., 2020). Preliminary human reports also suggest potential for mitigating cognitive decline by enhancing cholinergic signaling and reducing neuroinflammation (Jain et al., 2024).

Complementing these observations, sham-controlled studies in healthy adults indicate that nVNS enhances cognitive performance and resilience, with improvements in visuospatial reasoning, multitasking, sustained attention under sleep deprivation, reduced fatigue, and accelerated second-language learning in multi-day training contexts (McIntire et al., 2021; Klaming et al., 2022; Miyatsu et al., 2024).

Collectively, these findings suggest that nVNS may stabilize attentional and executive processes across a spectrum ranging from cognitive stress to early cognitive vulnerability. As this area advances, the development of objective biomarkers such as EEG, ERPs, and autonomic measures will be critical for defining dose–response relationships and identifying individuals most likely to benefit.

Chronic pain and central sensitization: While the strongest clinical data currently exist in primary headache disorders, mechanistic overlap with fibromyalgia, neuropathic pain, and complex regional pain syndrome suggests that vagal modulation could provide meaningful benefit in these syndromes (Russo et al., 2024).

Autonomic disorders: Early human studies demonstrate that cervical nVNS can improve heart rate variability and restore autonomic balance in conditions such as PTSD (Bremner et al., 2020). More recently, a preliminary study using auricular VNS reported positive effects in patients with postural orthostatic tachycardia syndrome (Stavrakis et al., 2024). Ongoing studies are evaluating the effects of cervical nVNS in POTS and related dysautonomias. Together, these findings point toward broader applications in dysautonomias in which vagal dysfunction plays a central role.

Gastroparesis: The vagus nerve plays a central role in coordinating gastric accommodation, antral peristalsis, and communication between the enteric nervous system and brainstem nuclei, making it a compelling target for neuromodulation in disorders of gastric motility. Early open-label human studies suggest that cervical nVNS may improve symptom burden in both idiopathic and drug-refractory gastroparesis, with reported benefits, particularly in nausea, fullness, and overall patient-reported symptom severity (Paulon et al., 2017; Gottfried-Blackmore et al., 2020). Importantly, these early studies consistently report good tolerability and an absence of serious device-related adverse events. While these findings are preliminary, they support further investigation into optimal stimulation parameters, dosing schedules, and patient phenotyping, as well as the relative contributions of central autonomic modulation versus direct effects on gastric motility.

nVNS and technology: Looking forward, integration of cervical nVNS with digital health technologies may enable adaptive, personalized dosing strategies. Closed-loop systems combining wearable physiological sensors with real-time neuromodulation could optimize stimulation parameters on an individual basis and expand access to bioelectronic therapies.

Optimization of stimulation delivery will likely involve modulation of temporal parameters such as frequency and duty cycle, which are known to influence patterns of neural recruitment, although within clinically used ranges these effects may be relatively broad rather than highly discrete (Han and Wang, 1992). In contrast, precise fiber-type selectivity remains inherently limited in transcutaneous approaches, as current spread through heterogeneous tissue preferentially recruits larger diameter fibers but does not allow for fine discrimination between afferent and efferent pathways.

Progress in stimulus optimization and delivery will depend on the development of mechanistic biomarkers, including functional imaging of brainstem and limbic activation, electrophysiological measures such as evoked potentials and EEG signatures, and peripheral markers of autonomic and inflammatory modulation, which may provide objective indicators of vagal engagement and guide individualized treatment. While closed-loop neuromodulation has been established in iVNS for epilepsy, where devices can respond to physiological signals such as heart rate to trigger stimulation (Winston et al., 2021), translation to non-invasive approaches will require identification of reliable, real-time physiological signals that can be feasibly monitored and integrated into adaptive stimulation paradigms. Recent work demonstrating fully automated, physiology-driven control systems further supports the feasibility of such approaches (Mathews et al., 2025).

Together, these directions highlight that while nVNS has already transformed the management of headache disorders, its broader therapeutic potential across immunology, psychiatry, pain medicine, and autonomic regulation is only beginning to be realized.

## 7 Conclusion

The convergence of evidence from randomized clinical trials, neuroimaging, electrophysiology, and immunology leaves little doubt that non-invasive cervical vagus nerve stimulation directly activates the vagus nerve. The physiologic, clinical, and biologic effects observed across studies cannot be explained by placebo responses and results from studies using sham stimulation rule out effects of nonspecific stimulation of cervical afferents (Tables 1, 2).

**TABLE 1:** Mechanistic and physiological evidence supporting vagal activation by cervical nVNS.

Domain	Key findings	Implication	References
Anatomy and biophysics	Cervical vagus lies superficially (∼1.3 cm); ∼80% of fibers are afferent. Modeling confirms cervical currents reach vagal depth with optimized parameters.	nVNS is anatomically and biophysically capable of recruiting vagal fibers.	Lerman et al., 2016; Groves and Brown, 2005; Mourdoukoutas et al., 2018.
Functional MRI	Activation of canonical vagal projection sites: NTS, locus coeruleus, insula, anterior cingulate.	Engagement of central vagal circuits.	Frangos and Komisaruk, 2017; Yakunina et al., 2017.
PET/molecular imaging	Altered connectivity in vagal–limbic networks; reduced neuroinflammatory markers with nVNS.	Central vagal modulation.	Wittbrodt et al., 2020.
Peripheral electrophysiology (eCAPs, vSEPs)	Conduction velocities consistent with Aδ, B, and C-fiber activation during nVNS; far-field vSEPs confirm vagal afferent recruitment.	Direct recruitment of vagal fibers with central transmission.	Bu et al., 2021; Nonis et al., 2017.
Cortical electrophysiology (EEG/ERP)	Enhanced N100 and P300b responses; effects lasting ≥ 2 h; absent with sham.	Objective cortical correlate of vagal afferent engagement.	Lewine et al., 2019.
Immunology	nVNS suppresses IL-1β, TNF-α, IL-6, IFN-γ and enhances IL-10 in healthy adults and patients; reduces CRP/procalcitonin in infection; improves symptoms and cholinergic signaling in autoimmune disease.	Activation of cholinergic anti-inflammatory pathway across systemic and neurologic disease.	Lerman et al., 2016; Brock et al., 2021; Tornero et al., 2022; Tarn et al., 2023.
Autonomics	nVNS increases HRV, suppresses sympathetic stress responses, improves vascular compliance	Real-time physiologic confirmation of vagal efferent engagement.	Brock et al., 2017; Bremner et al., 2020; Gurel et al., 2020.

**TABLE 2:** Clinical supporting vagal activation by cervical nVNS.

References	Population	Study design	Primary outcomes	Key findings
Gaul et al., 2016 (PREVA)	Adults with chronic cluster headache (*n* = 97 randomized; *n* = 93 ITT)	Prospective, randomized, open-label, controlled; cervical nVNS + standard of care vs. standard of care; preventive dosing: 3 × 2-min stimulations, twice daily	Change in weekly cluster headache attack frequency	Significant reduction in attack frequency vs. control (−5.9 vs. −2.1 attacks/week; Δ = 3.9; *p* = 0.02); higher ≥ 50% responder rate (40% vs. 8.3%)
Silberstein et al., 2016b (ACT1)	Adults with episodic and chronic cluster headache (*n* = 133 ITT; eCH *n* = 85, cCH *n* = 48)	Randomized, double-blind, sham-controlled trial of acute cervical nVNS vs. sham 3 × 2-min stimulations at attack onset, repeat allowed	Pain relief at 15 min (pain score 0–1) without rescue medication	No significant difference overall (26.7% vs. 15.1%; *p* = 0.1); significant benefit in episodic CH (34.2% vs. 10.6%; *p* = 0.008), not chronic CH
Goadsby et al., 2018 (ACT2)	Adults with episodic and chronic cluster headache (*n* = 92; eCH *n* = 27, cCH *n* = 65)	Randomized, double-blind, sham-controlled trial; 2-week treatment of acute attacks with cervical nVNS vs. sham 3 × 2-min stimulations at onset, repeat allowed	Pain-free at 15 min for treated attacks without rescue medication	No significant difference in total cohort (14% vs. 12%, *p* = 0.71); significant benefit in episodic CH (48% vs. 6%, *p* < 0.01) but not chronic CH
Silberstein et al., 2016a (EVENT)	Chronic migraine (*n* = 59; nVNS = 30, sham = 29)	Multicenter, randomized, double-blind, sham-controlled; preventive cervical nVNS vs. sham; 2 × 2-min stimulations, 2–3× daily (2 months), followed by open-label extension	Change in headache days per 28 days; safety/tolerability	No significant difference in randomized phase (−1.4 vs. −0.2 days; *p* = 0.56); significant reduction with extended use (−3.6 days; 95% CI −6.3 to −0.87); well tolerated with no serious device-related AEs
Tassorelli et al., 2018 (PRESTO)	Episodic migraine (*n* = 248 randomized; ITT *n* = 243)	Multicenter, randomized, double-blind, sham-controlled; acute cervical nVNS vs. sham; 2 × 2-min stimulations at onset (≤ 20 min), repeatable	Pain freedom at 120 min (first treated attack)	Primary endpoint not significant (30.4% vs. 19.7%; *p* = 0.067); significant at 30 min (*p* = 0.012) and 60 min (*p* = 0.023); repeated-measures significant (OR 2.3, 95% CI 1.2–4.4); improved pain relief
Diener et al., 2019 (PREMIUM)	Episodic migraine (*n* = 332 ITT; nVNS = 165, sham = 167)	Multicenter, randomized, double-blind, sham-controlled; preventive cervical nVNS vs. sham; 2 × 2-min stimulations, 3× daily (12 weeks)	Change in monthly migraine days	No significant difference vs. sham in ITT (−2.26 vs. −1.80 days; *p* = 0.15); sham device likely biologically active; significant benefit observed in adherent subgroup (≥ 67% adherence); well tolerated with no serious device-related adverse events.
Najib et al., 2022 (PREMIUM II)	Adults with episodic or chronic migraine (mITT *n* = 113; nVNS = 56, sham = 57)	Multicenter, randomized, double-blind, sham-controlled; preventive cervical nVNS vs. sham; 2 × 2-min stimulations, 3× daily for 12 weeks	Change in monthly migraine days	No significant difference in primary endpoint; higher ≥ 50% responder rate with nVNS (44.9% vs. 26.8%; *p* = 0.048); improved disability scores; greater effect in migraine with aura; well tolerated
Gurel et al., 2020	Adults with PTSD (*n* = 25; nVNS = 13, sham = 12)	Randomized, double-blind, sham-controlled; cervical nVNS vs. sham; 2-min stimulations delivered immediately after traumatic script exposure and mental stress (public speech, arithmetic), repeated across a 3-day protocol	Change in autonomic and physiological responses to stress	Reduced sympathetic responses to stress vs. sham (HR decreased, vascular tone increased); consistent attenuation of autonomic reactivity across stress paradigms
Bremner et al., 2021	Adults with PTSD (*n* = 20; nVNS = 9, sham = 11)	Randomized, double-blind, sham-controlled; cervical nVNS vs. sham; stress-paired 2-min stimulations followed by twice-daily self-administered treatment for 3 months	Change in PTSD symptoms (PCL, CAPS) and inflammatory response (IL-6) to stress	Significant reduction in PTSD symptoms vs. sham (greater reduction in PCL; *p* = 0.013); improved hyperarousal and somatic anxiety; blocked stress-induced increases in IL-6; well tolerated with no serious adverse events
Gazi et al., 2021	Adults with PTSD (*n* = 24; nVNS = 13, sham = 11)	Randomized, double-blind, sham-controlled; cervical nVNS vs. sham; 2-min stimulations following traumatic stress exposure	Change in cardiovascular responses to stress (reciprocal pulse transit time, 1/PTT)	Reduced stress-induced increases in 1/PTT compared with sham (*p* < 0.05), indicating attenuation of sympathetic vascular responses; effects observed during traumatic stress and early stimulation, with no differences under non-stress conditions
Choudhary et al., 2023	Adults with PTSD (*n* = 15; nVNS = 8, sham = 7)	Randomized, double-blind, sham-controlled pilot study; cervical tcVNS vs. sham; stimulation paired with cognitive tasks (paragraph encoding and *N*-back) during lab sessions, plus twice-daily home use over 3 months	Declarative memory (paragraph recall) and working memory (*N*-back performance)	Improved declarative memory (∼91% increase; *p* < 0.05); consistent trends in working memory improvement
Tornero et al., 2022 (SAVIOR I)	Hospitalized adults with COVID-19 (evaluable *n* = 97; nVNS = 47, standard of care = 50)	Prospective randomized controlled trial; cervical nVNS + standard of care vs. standard of care alone; 2 × 2-min stimulations, 3× daily (bilateral) during hospitalization	Change in inflammatory biomarkers (CRP, procalcitonin) and clinical outcomes	Significant reductions in CRP and procalcitonin vs. control; no difference in respiratory outcomes; no serious device-related AEs

Concerns about current penetration have been systematically addressed: electrophysiological measures such as event-related potentials provide direct evidence of vagal fiber engagement, neuroimaging demonstrates activation of canonical vagal projection sites, and cytokine studies confirm recruitment of the cholinergic anti-inflammatory pathway. Together, these findings provide a mechanistic fingerprint unique to vagal activation, eliminating alternative explanations.

In keeping with the Hippocratic principle *primum non nocere* (“first, do no harm”), non-invasive strategies should be prioritized whenever possible. While iVNS will remain valuable, particularly for refractory conditions, cervical nVNS offers a safe, effective, economical, and durable means of engaging vagal physiology. By analogy to peripheral nerve stimulation, its distant yet powerful central effects may represent not a limitation, but an advantage.

nVNS thus stands as a model of bioelectronic medicine—illustrating how precise peripheral stimulation can reshape central physiology, reverse maladaptive states, and deliver lasting therapeutic benefit.
